# Gamified online surveys: Assessing experience with self-determination theory

**DOI:** 10.1371/journal.pone.0292096

**Published:** 2023-10-13

**Authors:** Alice H. Aubert, Andreas Scheidegger, Sara Schmid

**Affiliations:** 1 Eawag: Swiss Federal Institute of Aquatic Science and Technology, Dübendorf, Switzerland; 2 Institute of Natural Resource Sciences, Zurich University of Applied Sciences, Wädenswil, Switzerland; Shandong University, CHINA

## Abstract

We developed four online interfaces supporting citizen participation in decision-making. We included (1) learning loops (LLs), good practice in decision analysis, and (2) gamification, to enliven an otherwise long and tedious survey. We investigated the effects of these features on drop-out rate, perceived experience, and basic psychological needs (BPNs): autonomy, competence, and relatedness, all from self-determination theory. We also investigated how BPNs and individual causality orientation influence experience of the four interfaces. Answers from 785 respondents, representative of the Swiss German-speaking population in age and gender, provided insightful results. LLs and gamification increased drop-out rate. Experience was better explained by the BPN satisfaction than by the interface, and this was moderated by respondents’ causality orientations. LLs increased the challenge, and gamification enhanced the social experience and playfulness. LLs frustrated all three needs, and gamification satisfied relatedness. Autonomy and relatedness both positively influenced the social experience, but competence was negatively correlated with challenge. All observed effects were small. Hence, using gamification for decision-making is questionable, and understanding individual variability is a prerequisite; this study has helped disentangle the diversity of responses to survey design options.

## 1. Introduction

### 1.1. General motivation

Many fields such as environmental and public health care sciences seek to engage citizens, including laypeople, in complex decision-making processes [e.g., 1–3]. How to best engage citizens in complex public decision-making is still unclear, but relying on information and communication technology appears to offer substantial promise [4, 5]. For instance, e-negotiation platforms [6, 7] and specifically designed online surveys [8–10] have been developed and tested. Our research contributes to this endeavor: We developed a novel online survey to collect citizens’ preferences in complex structured decision-making supported by multicriteria decision analysis (MCDA).

Some researchers are concerned that without guidance from an experienced decision analyst, the citizens may be overwhelmed by often required tedious and repetitive tasks [11]. Consequently, they fear that data collected through these online interfaces would be too unreliable to support public decision-making. Studies on surveys and survey design also stress that long and cognitively demanding surveys lead to *satisficing*, a portmanteau term combining *satisfy* and *suffice* [12]. Examples of satisficing behaviors include speeding through the survey and not differentiating among objects in rating, termed straightlining [12]. In extreme cases, respondents simply abandon the survey, termed drop-out [13]. Survey scientists have warned that online tools and increased solicitations may render respondents even more prone to such satisficing behaviors [14, 15]. Therefore, a form of gamification recently termed “surveytainment” [16] has been explored to support online survey quality [14, 15]. The innovation of our study is to gamify online surveys for participatory decision-making to obviate any assistance from a decision analyst.

Gamification originates from information and communication technology [17] and uses elements of game design in nongame contexts to enhance a service or product [18, 19]. Gamification is often used to increase users’ participation and performance in online computer interactions [e.g., 20–23]. Research on online survey design has also tested the effectiveness of gamification, which supposedly offers an enjoyable experience, to retain respondents in long surveys [14, 15, 24, 25]. We investigated whether gamifying our survey interface for participatory decision-making influenced the drop-out rate and the respondents’ experience.

Our study aimed to overcome some limitations observed in the literature. Gamification is rarely rigorously evaluated (e.g., studies often lack a control treatment) and is still most often referred to only as potentially promising [17, 26]. The surveytainment literature has thus far limited measures of the enjoyment of the survey to self-reported recommendation to others, and first results do not report lower drop-out rates [15]. Therefore, we designed our study to measure more facets of users’ survey experience. In addition, this study complemented previous ones [27–29] with an improved experimental design, interfaces designed newly in response to feedback we had collected about previous interfaces, and a full-scale decision problem. One major improvement in the experimental design reported here is that we can differentiate the effects of gamification from those of learning loops. Learning loops provide the user with feedback on the consistency of preferences elicited in two ways. They may be received as an annoyance or welcomed as a challenging opportunity to learn. Our experiment included control treatments, a relatively large sample with 785 observations representative of German-speaking Swiss population in age and gender, and measurement instruments retrieved from the literature. It thus overcomes limitations found in many gamification studies.

**Fig 1:**
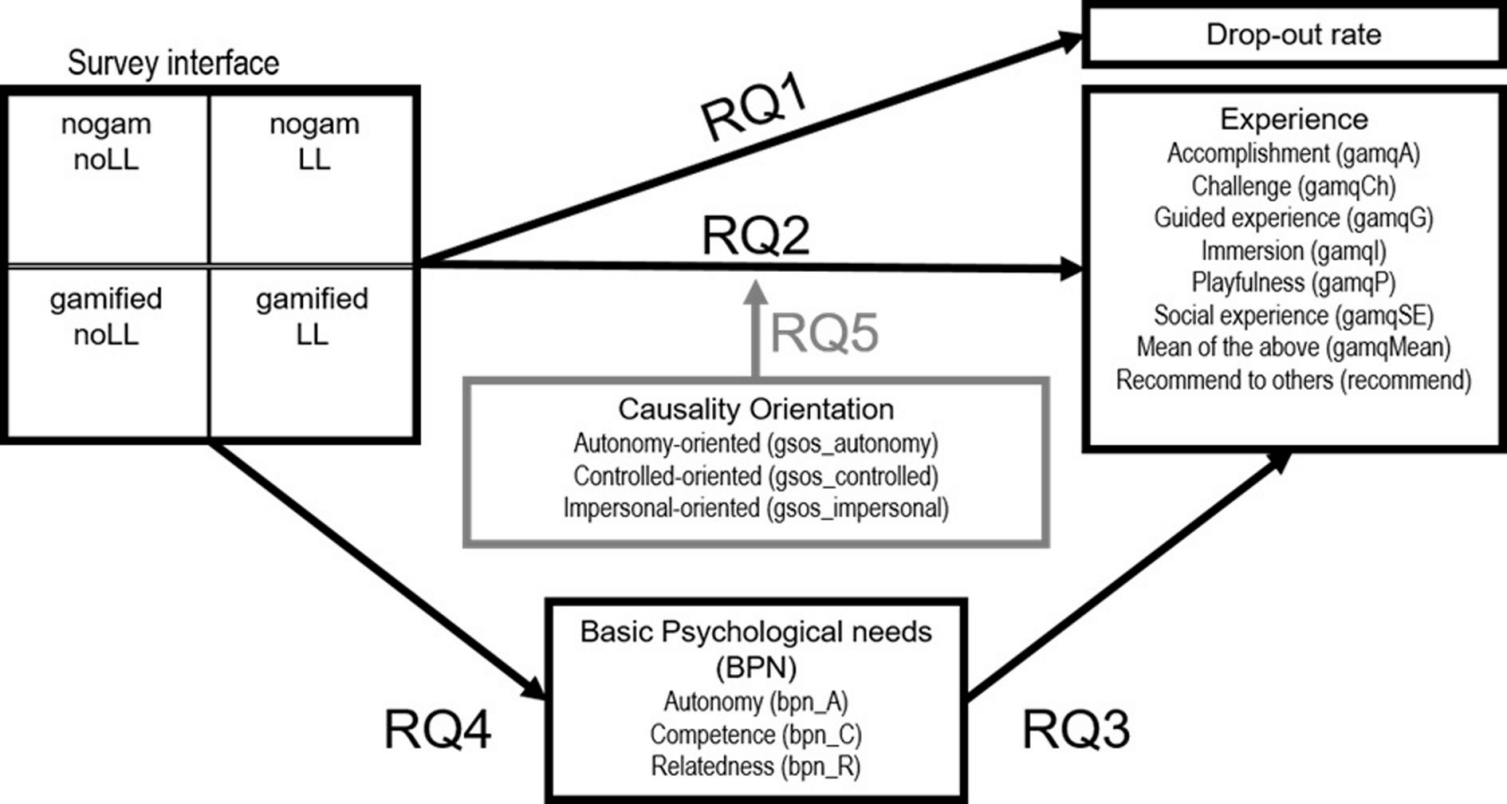
Summary of the research questions (RQ). Nogam: nongamified. noLL: without learning loop. LL: with learning loop.

The self-determination theory, commonly used in gamification studies [30], provides a good framework for our research questions (Fig 1, Section 1.2). We designed an experiment (Section 2) to test whether and how gamification and learning loops influenced the survey experience. Our results (Section 3) were based on a relatively large sample. Results are discussed in Section 4.

### 1.2. Theory and background

#### 1.2.1. Retention of respondents (RQ1)

As mentioned in Section 1.1, one of the issues reported about gamified survey is that they lower the response and completion rates [15, 31]. For instance, in Harms, *et al*. [32], the response rate for the gamified survey was lower (70%) than for the control (86%). In Guin, Baker, Mechling and Ruyle [14], the participation rate was of 8%; the completion rate was 72% for the gamified survey but 93% for the other treatments. Although gamification is said to be engaging and should retain respondents, it seems that it is not the case in all circumstances. Unsurprisingly, more difficult and longer surveys lead to higher drop-out rates [31, 33]. Because our online survey for decision-making was very long and somewhat tedious and repetitive, particularly when learning loops were included, we sought ways to retain respondents by testing gamification. We would like to identify the degree to which gamification and learning loop influence the drop-out rate. This leads to our first question:

RQ1: How does the online survey interface influence the drop-out rate for our long and complex case?

#### 1.2.2. Experience (RQ2 to 4)

Studies on gamification from information and communication technology commonly refer to the self-determination theory (SDT), and in particular the basic psychological needs (BPNs) subtheory [30, 34–37]. It suggests that any factor satisfying the three basic psychological needs of autonomy, competence, and relatedness improves functioning, including performance and persistence at tasks (quote of original proposition in Supplementary Material (S1 File) S1 Sec). Game elements such as rewards and feedbacks are external events that can have informational functional significance by affirming or promoting the basic needs of autonomy, competence, and relatedness [38].

Autonomy is the need to act with a sense of choice and volition. A common way to satisfy the need for autonomy is to provide choices [36, 39, 40] or nonfixed structure [37]. Immersion-related game elements such as narrative, role-play mechanics, and customization create meaningful storylines and satisfy the need for autonomy [41]. Competence is the need to be effective and master tasks. Competence can be satisfied by providing clear goals and unlocking the next difficulty level when easier levels are achieved [36]. Competence can be frustrated when tasks are not adapted to the player, for instance when they are too difficult or too easy [42]. Relatedness is the need to be socially connected. The need for relatedness can be satisfied through social networks, or in single-user interfaces through interactions with nonplayer characters [36]. Competition and cooperation can also satisfy the need for relatedness [37, 41]. By enhancing the basic needs satisfaction and lowering their frustration, gamification should lead to performance that is more effective and to persistence at difficult and complex actions, and a more positive experience. Several studies on human-computer interactions have reported positive effects of gamification on the three constructs of the BPN theory [37, 41], but to the best of our knowledge, no studies of surveytainment have yet done so. We ask the following research questions (RQ):

RQ2: How does the online survey interface influence experience? We expect that a gamified survey including choices, small tasks and rewards, and interactions with nonplayer characters would improve the experience. However, this effect could be counterbalanced if the survey is too difficult, for instance by including a task that is difficult to resolve. In our case, this difficult task to resolve consists in a so-called learning loop, where participants are shown their own answers elicited from two different methods and are asked to resolve the inconsistencies, if observed.

RQ3: Can the basic psychological needs theory explain experience? We expect that high needs satisfaction improves the experience.

RQ4: Does the survey interface influence the satisfaction of basic psychological needs? We expect that a gamified interface satisfies the basic psychological needs better. However, if the survey is too difficult, for instance because it includes a learning loop, the needs, particularly for competence, could be frustrated.

#### 1.2.2. Individual characteristics (RQ5)

Previous studies suggest that respondents’ individual characteristics can moderate the experience. For instance, women reported greater social benefits from using a gamified service for health than men [43]. The same study also showed that the ease of use of this gamified service for health declined with age [43]. Recently, the Big Five personality traits were also studied: High neuroticism enhanced the increase of enjoyment that gamification created [44]. Some have commented that previous studies referring to SDT oversimplify the theory by considering only the basic psychological needs subtheory and ignoring the other subtheories [38, 45]. Among several suggestions, Loughrey and Broin [45] invite follow-up research investigating individuals’ causality orientations. They propose to verify whether individuals that perceive more external regulation, termed controlled-oriented or impersonal-oriented individuals, are more likely to react positively to extrinsic motivational elements such as game elements, as opposed to individuals perceiving more internal regulation, who are termed autonomy-oriented individuals [38, 45]. This was first researched by Mekler, Brühlmann, Tuch and Opwis [20]. The original description of individuals high in autonomy orientation is that they are “likely to display greater self-initiation, seek jobs that are interesting and challenging and take greater responsibility for [their] own behavior” [46]. Individuals high in controlled orientation “are likely to be dependent on rewards or other controls, and may be more attuned to what others demand that to what they want for themselves” [46]. Finally, individuals high in impersonal orientation “have no sense of being able to affect outcomes or cope with demands or changes”, “attaining desired outcomes is beyond [their] control and … largely a matter of luck or fate” [46]. This leads to our fifth research question:

RQ5: Does respondents’ general causality orientation predict how the survey interface is experienced? We expect that autonomy-oriented respondents have a positive experience independently of the survey interface. In contrast, we expect that controlled- and impersonal-oriented respondents have a more negative experience, particularly if the survey is difficult, for instance because it includes a learning loop.

To answer the five research questions (Fig 1), we designed an experiment with control treatment.

## 2. Methods

### 2.1. The survey

We developed an online survey to elicit preferences from citizens for decisions supported with multicriteria decision analysis (MCDA) [27, 47]. Our online interfaces collect the relative importance, termed weights that citizens give to the various objectives that the decision affecting them has to achieve. Because these objectives cannot all be achieved concurrently, trade-offs between objectives are necessary. The weights represent trade-offs between objectives. We followed standard swing and trade-off methods for weight elicitation, which are somewhat complex and repetitive [e.g., 48]. Good practice in the decision analysis field recommends that consistency check questions be implemented [49]. We did so for half of the respondents, even though it made the survey even longer. We refer to these consistency checks as learning loops, because they create a cognitive dissonance that should trigger reflection and change respondents’ mental models: They should trigger learning [50]. The length of survey, estimated between 45 and 60 minutes, is far longer than recommended for surveys in market research. Good practice is to ask 20 questions (per stage if multistage) for a maximum survey duration of 13 minutes [33]. However, this recommendation may not need to be followed strictly if respondents consider the survey relevant [14]. We thought that offering a more engaging experience through gamification could help. The gamified and nongamified treatments are presented in Section 2.2.1 and S3 Sec (S1 File) and, the treatments with and without learning loop in Section 2.2.2.

### 2.1. The experiment

**Fig 2:**
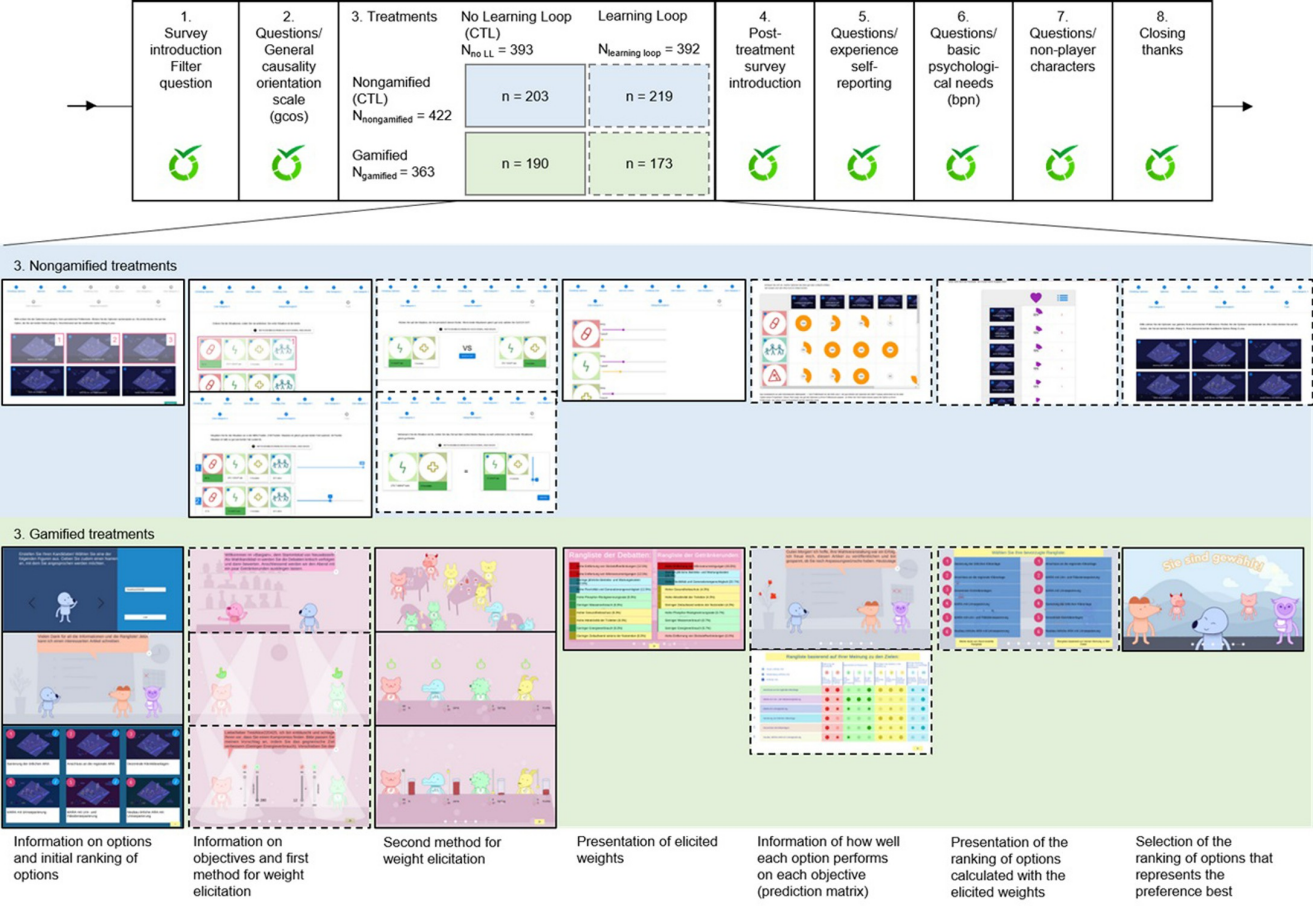
**The experiment and sample sizes** (after data cleaning) (top line). CTL: control treatment; LL: learning loop. **Screenshots of treatment interfaces** (middle line: nongamified treatments; bottom line: gamified treatments). Screenshots enclosed in a solid line were included in the treatments without and with learning loops; screenshots enclosed in a dashed line were included only in the treatments with learning loops.

We created a gamified version of the weight elicitation survey by Aubert and Masson [47]. Weight elicitation is one step of a multicriteria decision analysis process. It consists in asking respondents for their preferences about the relative importance of objectives that cannot all be achieved at the same time. Weight elicitation corresponds to preferences in how respondents handle trade-offs in a complex decision. Because we expected that the learning loop could affect the experience, we created two gamified versions. We designed a 2 × 2 between-subject experiment, with the varying factors: gamified vs. nongamified (control) and with vs. without (control) learning loop. Consequently, we had four treatments (Fig 2).

Fig 2 shows the flow of the experiment. After clicking on the link from the invitation email, respondents were welcomed with a short introduction to the public decision at stake, wastewater management, and the reasons why this topic matters in rural Switzerland. Then, they answered a filter question. We targeted laypeople, and therefore filtered out respondents that knew *rather a lot* or *a lot* about wastewater management. Respondents knowing *nothing at all* to *a little* could proceed to answer the general causality orientation scale (Section 2.4). After that, respondents were automatically directed to one of the four treatments. Respondents read about ten objectives that wastewater management needs to achieve and six possible alternatives based on Swiss data from a case study [51]. After informing them, we elicited their preferences by asking them to weight the objectives. Thereafter, they were automatically directed to the post-treatment questionnaire, which included an introduction, the gamefulquest scale to measure experience [52], the basic psychological needs satisfaction and frustration scale to measure autonomy, competence, and relatedness, questions about the nonplayer characters (Section 2.4), and a thank you.

#### 2.2.1. Gamification: With vs. without (control)

The nongamified control treatment was a survey interface, specifically designed to elicit the weights in MCDA. The interface followed guidelines from the decision analysis field [49, 53] by providing information on the context and elements of the decision. The elicitation part of the survey used state-of the art methods to focus on how people make trade-offs. The nongamified interface resulted from iterative development: Two prototypes had been developed and tested before [29, 54]. The nongamified interface of the present study had a simple design displaying a progress bar. Instructions were improved, accessible on demand, and included illustrated examples. The interface provided pop-up warning messages in some cases. For screenshots, see S4.3 Sec in S1 File.

The treatment with gamified interface provided exactly the same information on the context and elements of the decision. The methods for the weight elicitation were the same, although simplified in some cases and adapted to the narrative (S4.2 Sec in S1 File). We gamified the weight elicitation by adding a challenging narrative which provided a motive, rewards and progress through the chapters, a choice of avatar, guidance from some nonplayer characters, interactions with other nonplayer characters, a specific visual design, and ambient sound. Respondents could make some more choices in the gamified treatment than in the control. Adding these game elements should satisfy the three BPNs of the self-determination theory (Table 1).

**Table 1:** Game elements included in the gamified treatments and the basic psychological needs they target [e.g., 36].

Game element / players’ actions	Targeted needs
Choosing an avatar	Relatedness, autonomy
Progressing through the story	Competence
Interacting with nonplayer characters	Relatedness
Being guided by some nonplayer characters, receiving feedback from them	Relatedness
Map / moving around New Waterton	Autonomy
Moving around in the bar (selecting the table)	Autonomy
Answering small challenges with clear goals	Competence
Being elected: reward	Competence

The gamified interface should immerse the respondents in a story. The narrative was that the respondents were candidates to be mayor of their town, New Waterton. A scandal related to deficient wastewater management in the neighboring town highlighted the topic of wastewater management in New Waterton. The citizens of New Waterton made wastewater management decisive for their vote: They would consider the players’ consistent position on this topic when electing the mayor. At the start, a journalist and a wastewater engineer approached the players. The journalist aimed to write an article informing the citizens about the players’ preferences on wastewater management. The engineer explained the context and presented six management options suitable for New Waterton. The players ranked these options for the article. The players asked the journalist to check the article before publication after having met with citizens. Later in the day, the players met ten citizens from New Waterton. Each citizen advocated one objective. In the evening, a campaign meeting took place in the local bar. The ten advocating citizens challenged the players by asking their positions on the various objectives. At first, this was done by verbal jousting. As the evening went on, advocates sat at their tables for rounds of drinks. Before closing the bar, the bartender summarized the campaign meeting. This informed the players about the weights elicited. The next day, the players met the journalist and the engineer again to check the article. They were informed about the results of the campaign meeting, and the engineer also presented a ranking of options based on the preferences discussed at the bar. Players chose which ranking should be in the article. In the closing chapter, the players were informed that they were elected as the new mayor of New Waterton. For screenshots, see S4.1 Sec in S1 File.

#### 2.2.2. Learning loop: With vs. without (control)

The control treatment without learning loop was a linear survey comprising three steps: (1) information on management options and initial ranking of options, (2) information on objectives, weight elicitation with the swing method [see e.g., 48], and presentation of the weights elicited, and (3) a final ranking of option (Fig 2). The narrative of the gamified treatment was adapted accordingly.

The treatment with learning loop included elicitation with two methods and comparison of the results elicited (Fig 2). This comparison of the results from two methods constituted a consistency check. We had tested the concept of our consistency check previously [27]. It was successful because it did not judge respondents’ preferences but provided them with an additional opportunity to consider how they weighted the objectives, and ranked the options. The learning loop differed slightly in the nongamified and gamified treatments (Fig 2) in the order of the methods and when the consistency check occurred.

### 2.3. Sample definition and recruitment

Respondents were German-speaking Swiss adults, selected by a market research company (www.intervista.ch, retrieved on 20.6.2022). Intervista was contracted to ensure that the sample was representative of Swiss population statistics in age and gender (S5 Sec in S1 File) and covered all education levels equally in each treatment. Intervista invited respondents by sending an email with a link to the opening survey. Intervista ensured that respondents participated only once. Intervista informed them about the unusually long survey (45 to 60 minutes) and the requirement to answer the survey on a desktop or laptop computer. By proceeding to the survey, respondents gave their consent to participate. They were informed of their rights to stop and withdraw their answers, anonymity was ensured. Authors had no information to identify the respondents. Upon completion, respondents received points according to the company’s incentive system. Based on previous work [55] and a priori statistical power analysis, we aimed at 200 respondents per treatment. Data for the nongamified treatments were collected between March and April 2021 and for the gamified treatments between October and November 2021. The experiment was part of a project which underwent ethical review and was evaluated as “minimal risk project involving human subjects”.

### 2.4. Measurement instruments

Table 2 describes the measurement instruments in the order in which the respondents answered them (Fig 2). The questions were coded on the LimeSurvey platform. The full questionnaire is available in the Supplementary Material (S6 Sec in S1 File). We adapted items of some scales, including the BPN satisfaction and frustration scale [56] and the Gamefulquest scale [52]. This is a common practice in studies on gamification [e.g., 35, 39–41, 57, 58] because the items need to be adapted to the specific experiment. We retained as much of the original wording as possible, only modifying it slightly to match the tasks of our treatments. The scales were translated from English to German and back-translated. Problematic items were discussed with peers. The experiment was pretested with six respondents by Intervista with think-aloud protocols and adjusted as necessary. All Cronbach’s alpha were showing at least acceptable reliability (>0.7, see S7.1 Sec in S1 File) over the items of a single construct.

**Table 2:** Measurement instruments used.

	Measurement instrument	Ref. & SI
**1. General causality orientation (gcos)**	Original short version of the general causality orientation scale. It consisted of 12 vignettes, each including three types of reactions (autonomy-, controlled- and impersonal-oriented), making a total of 36 items, answered on 7-point Likert scales, from 1 (very unlikely) to 7 (very likely). Each respondent was characterized by the sum score on the three dimensions of orientation (autonomy, controlled, and impersonal: gcos_autonomy, gcos_controlled, gcos_impersonal). Each varied between 12 and 84.	Deci and Ryan [59]
**2. Overall experience (Gamq)**	• Gamefulquest scale for the experience of gamification (partly validated). We used the items related to the following six constructs: accomplishment (8 items), challenge (8 items), guided experience (7 items), immersion (9 items), playfulness (9 items), and social experience (8 items). Due to the length of survey, we removed the items for the construct of competition (not a feature of our gamification). We also removed some items in the six constructs considered when their adaption to our context would have been too strong. Respondents scored statements on 7-point Likert scales, from 1 (not at all true) to 7 (totally true). We calculated the mean for each construct (Gamq_A, Gamq_Ch, Gamq_G, Gamq_I, Gamq_P, Gamq_Se) and, the overall mean (Gamq_Mean).	Högberg, Hamari and Wästlund [52]
	• We also adapted items on the recommendation of the survey to others. There were 5 items. Respondents scored statements on 5-point Likert scales from 1 (not at all true) to 5 (totally true). We calculated the mean (recommend).	Harms, Wimmer, Kappel and Grechenig [32], Sheldon and Filak [39]
**3. Basic psychological needs satisfaction and frustration (bpn)**	Training domain-specific items of the BPN satisfaction and frustration scale. We used items related to the three constructs: autonomy (8 items), competence (8 items), and relatedness (8 items). Respondents scored statements on 5-point Likert scales from 1 (not at all true) to 5 (totally true). We calculated the mean for satisfaction items minus the mean for frustration items for the three constructs (bpn_A, bpn_C, bpn_R). It varied between -4 and 4.	Gagné [56]
	Note, following results for our previous experiment, where we had observed that relatedness in gamified survey on societal topic was twofold, we focused on a single aspect, relatedness towards fellow Swiss citizens who are facing decision on wastewater management.	Aubert, Lienert and von Helversen [28]
**4. Perception of the nonplayer characters**	5-point Likert question: “How much did you like the nonplayer characters (graphical presentation, characteristics, etc.)?” from 1 (not at all) to 5 (very much). We completed the exploration of the perception of nonplayer characters with an optional long textbox for respondents who liked “a bit” to “very much” the nonplayer characters: “Please elaborate what you have liked, resp. not liked, about the nonplayer characters”.	-

### 2.5. Data analysis

We represented the research questions 2 to 5 in linear regression models as presented in Section 3. Residual diagnostics showed that the model assumptions were met for all models. All statistical analyses were performed in R [60]; the code and data are available (https://doi.org/10.25678/0008VR).

We coded the qualitative data as negative, positive, negative and positive, neutral, or unclear. We described the perception of the nonplayer characters by the adjectives used in the comments. The respondents provided comments in their first language, German, and we translated those that appear in this paper and S1 File.

## 3. Results

### 3.1. Respondents (RQ1)

The first phase of the experiment (step 1 in Fig 2) was accessed by 2446 respondents (distribution per treatment in Table 3). Only 36% (881 respondents) proceeded to the end of the post-treatment questionnaire. This high drop-out rate (64%, Table 3) can be explained both by the length of survey and by a technical issue that arose with the gamified treatment: The hosting server crashed during data collection, preventing some respondents from proceeding in the survey. Some respondents also emailed us that they could not complete the survey. The reasons were diverse: In the absence of a back button, they clicked on their browser’s back button, which led them to the opening survey that they had already completed, thus indicating that the session had expired. Despite the instruction in the invitation email to answer the survey only from a desktop or laptop, many tried to answer the survey on a tablet or a smartphone. Some respondents also wrote that choosing an avatar and a name “confused” them, and it appeared to them as “unsafe and not serious”, so they stopped. We removed 96 respondents who satisficed by straightlining and/or speeding. Our final sample contains 785 observations (Fig 2 and Table 3). Age, gender, and education distributions are similar across the subsamples (S5 Sec in S1 File). We investigated if the individual causality orientation scores related to drop-out. We found only a small effect: Respondents with higher controlled-orientation are more likely to drop-out (S5 Sec in S1 File). We discuss potential consequences in the result sections below (Section 3.3).

**Table 3:** Statistics of respondents. Start: number of respondents who accessed the opening part of the survey; Complete: number of respondents who completed the survey until the end; Sample: number of respondents after data cleaning (removing straightlining, speeding), %Lost: proportion of respondents lost from the starting sample. LL: learning loop. nogam: nongamified.

	Start		Complete		Sample		% Lost	
	**noLL**	**LL**	**noLL**	**LL**	**noLL**	**LL**	**noLL**	**LL**
**nogam**	431	614	224	243	203	219	52.9	64.3
**gamified**	571	830	209	205	190	173	66.7	79.2

### 3.2. Experience of the interface and basic psychological needs (RQ2 to RQ4)

#### 3.2.1. Interface to experience (RQ2)

**Fig 3:**
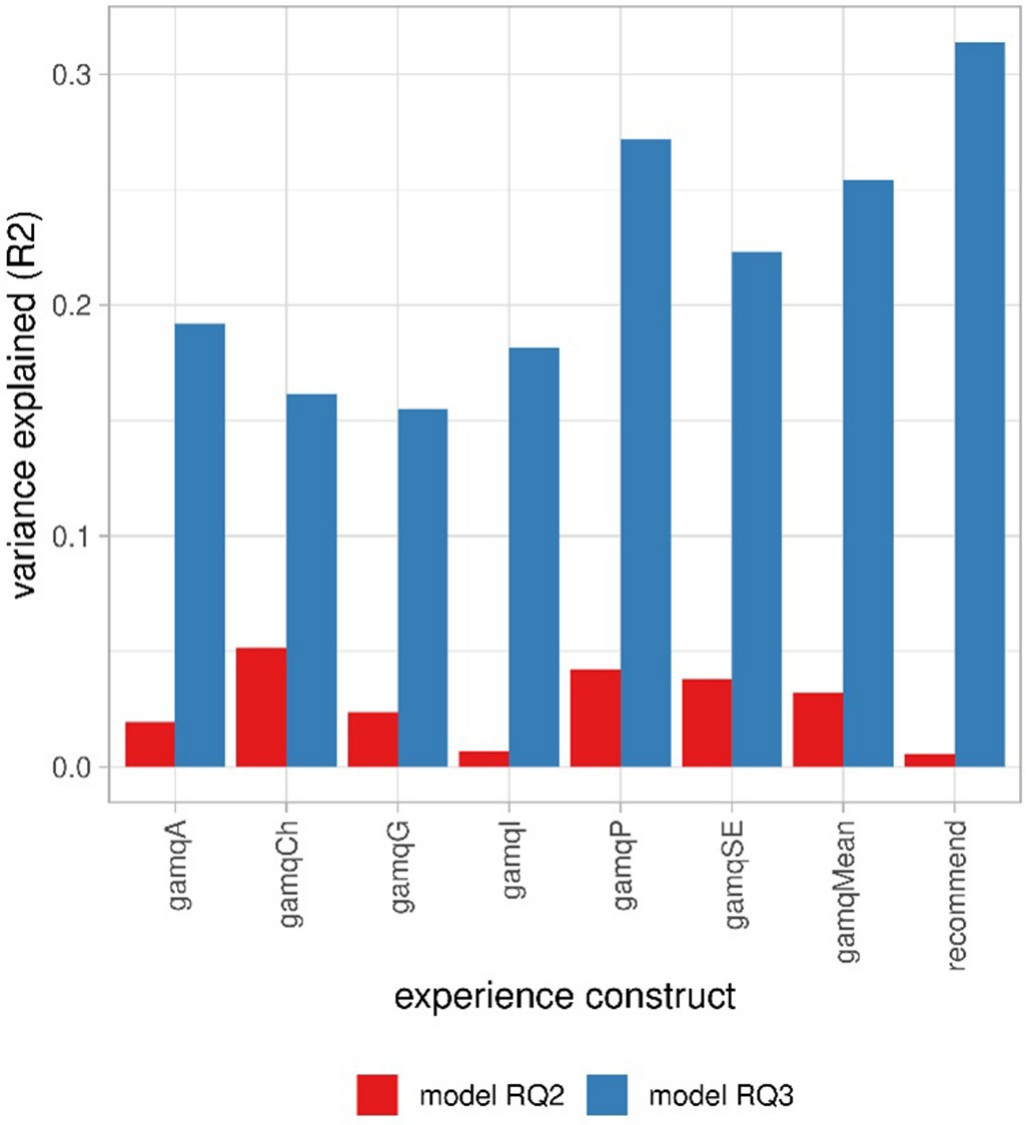
Variance explained (R^2^) for the constructs of experience by the interface (model RQ2, red left bars) and by the basic psychological needs (model RQ3, blue right bars). gamqA: accomplishment. gamqCh: challenge. gamqG: guided experience. gamqI: immersion. gamqP: playfulness. gamqSE: social experience. gamqMean: mean of the previous six constructs. recommend: recommendation of survey to others.

**Fig 4:**
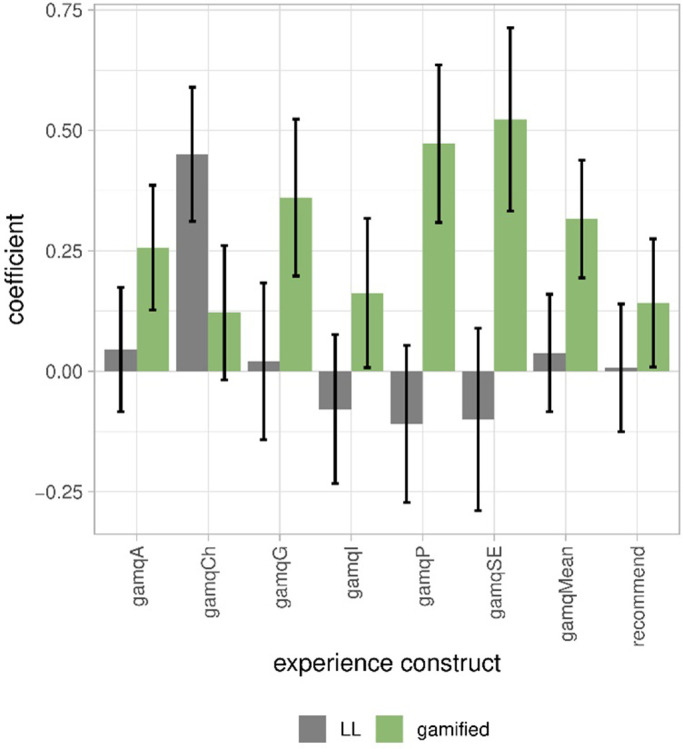
Coefficients of the factors gamification and learning loop (LL) explaining the constructs of experience and 95% confidence intervals. gamqA: accomplishment. gamqCh: challenge. gamqG: guided experience. gamqI: immersion. gamqP: playfulness. gamqSE: social experience. gamqMean: mean of the previous six constructs. Varied from 1 to 7. recommend: recommendation of survey to others; varied from 1 to 5.

Overall, for most constructs, respondents rated their experience as neutral (Tab.S721 in S1 File). Regression models were constructed to predict the different constructs of experience (gamq scores) with the factors gamification and learning loop (LL). All modes have a very small explanatory power: from 1% variance explained for immersion to 5% for challenge (Fig 3, Tab.S723 in S1 File). Gamification has very small effect on the following constructs of experience: accomplishment, guided experience, playfulness, and social experience (Fig 4, Tab.S722 in S1 File). The learning loop slightly increased the perceived challenge (0.45 points, *p* < 0.001). Overall, the interfaces explained little of the experience.

#### 3.2.2. Basic psychological needs to experience (RQ3)

**Fig 5:**
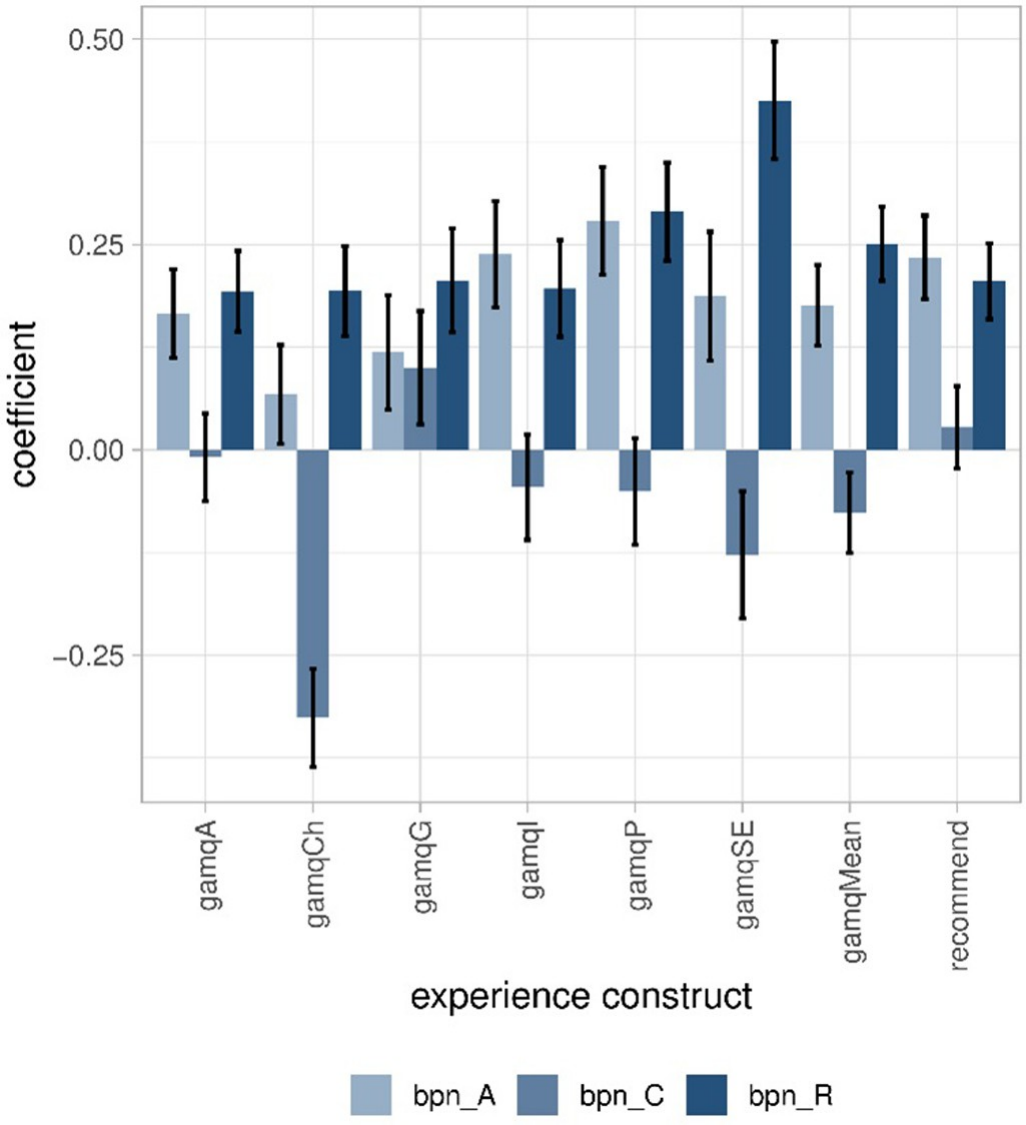
Coefficients of autonomy, competence, and relatedness explaining the constructs of experience with 95% confidence intervals. gamqA: accomplishment. gamqCh: challenge. gamqG: guided experience. gamqI: immersion. gamqP: playfulness. gamqSE: social experience. gamqMean: mean of the previous six constructs; varied from 1 to 7. recommend: recommendation of survey to others; varied from 1 to 5. bpn_A: autonomy. bpn_C: competence. bpn_R: relatedness.

We investigated whether the three constructs of the BPN satisfaction and frustration scale were better predictors of the experience than the interface. The three needs of autonomy, competence, and relatedness were on average satisfied rather than frustrated (means > 0) (Tab.S731 in S1 File). For the regression models predicting the constructs of experience from autonomy, competence, and relatedness, the variance explained ranged from 15.4% of the variance for guided experience to 31.4% for the recommendation to others, an improvement compared to the models using the interface (see Fig 3 for a comparison). Autonomy had the most positive effect (above 0.12 points) on accomplishment, immersion, playfulness, social experience, the mean of the experience constructs, and the recommendation to others (Fig 5, coefficients and *p* values in Tab.S732 (S1 File)). Relatedness exhibited a similar pattern of positive effect on social experience, playfulness, accomplishment, challenge, guided experience, immersion, the mean of the experience constructs, and recommendation to others (Fig 5, Tab.S732 in S1 File). Competence had a completely different pattern. In particular, feeling competent significantly reduced the experience of challenge (Fig 5) and social experience. However, feeling competent proved to have a positive effect on perceiving a guided experience. The effect of competence on the mean of the experience constructs was almost null. The BPNs were stronger predictors of experience than the interface. This could be due to personality traits and is investigated in RQ5. Feeling related and autonomous had a positive effect.

#### 3.2.3. Interface to basic psychological needs (RQ4)

**Fig 6:**
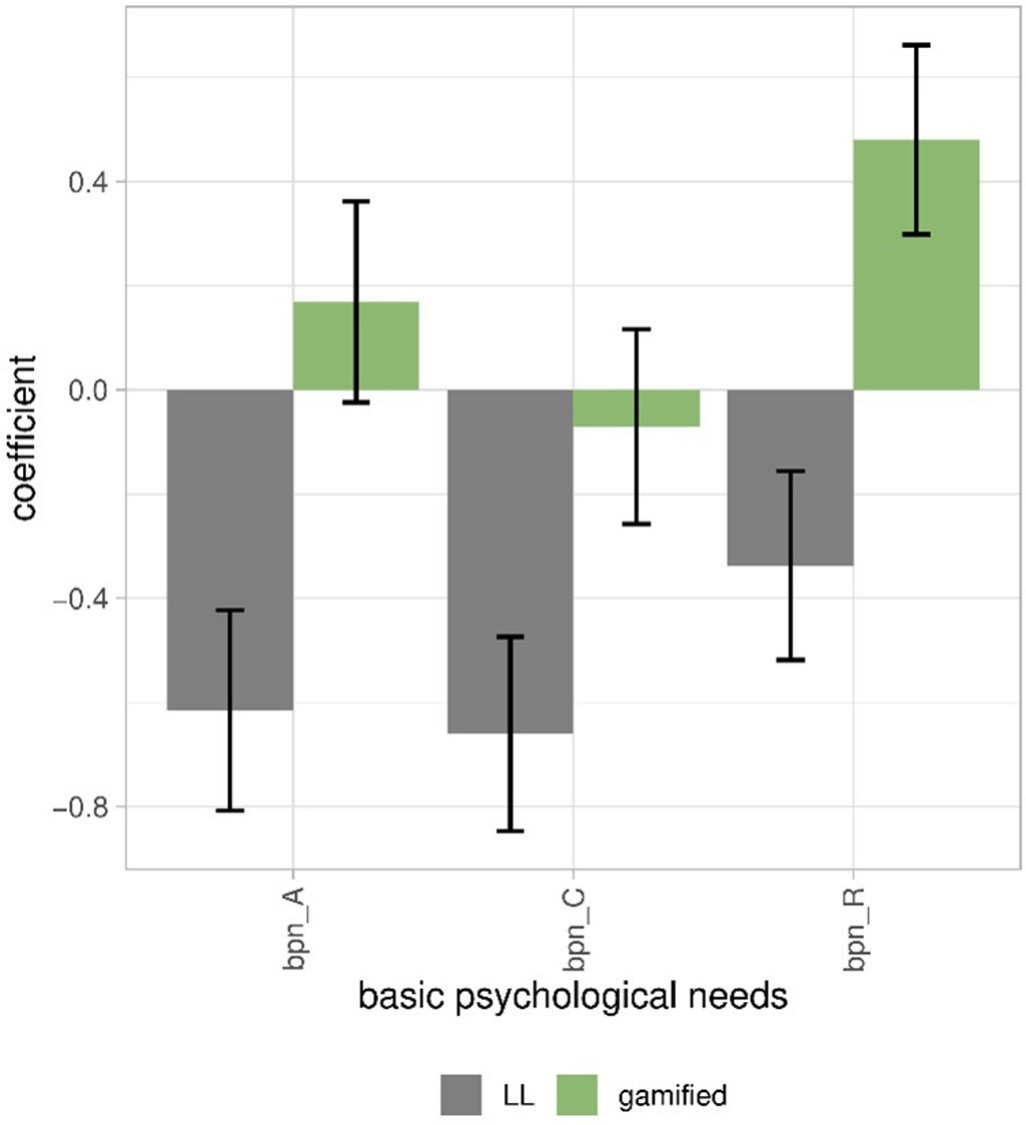
Coefficients of the two factors of interface, gamification and learning loop, explaining the constructs of basic psychological needs and 95% confidence intervals. bpn_A: autonomy. bpn_C: competence. bpn_R: relatedness. Varied from -4 to 4.

Finally, we investigated how much the interface had an effect on the BPN satisfaction and frustration. For each need, we fitted a regression model with gamification and learning loop as inputs. The models explained only 5 to 6% of the variance (R^2^_bpn_A_ = 5.3%, R^2^_bpn_C_ = 5.8%, R^2^_bpn_R_ = 5.1%). Nevertheless, we obtained a few significant parameters: Gamification had a positive effect on relatedness and to a lesser extent on autonomy (Fig 6, coefficients and *p* values in Tab.S741 (S1 File)). In contrast, the learning loop had a negative effect on competence, autonomy, and relatedness (Fig 6, Tab.S741 in S1 File). These small effects follow our expectation: The gamification with a narrative including guidance and feedback from nonplayer characters increased the relatedness need satisfaction, and the learning loop frustrated all three needs.

### 3.3. Individual causality orientation and experience of interface (RQ5)

**Fig 7:**
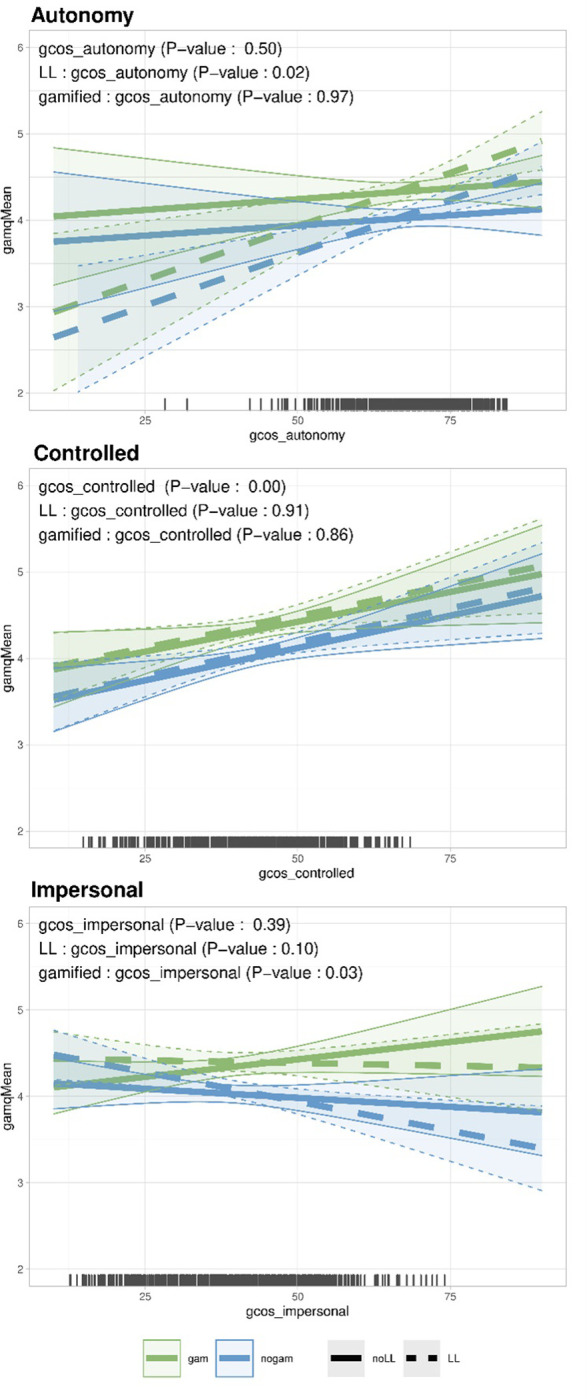
Predicted mean experience (gamqMean) with 95% confidence intervals as a function of the autonomy (top panel), controlled (middle panel), and impersonal orientation (bottom panel). All three panels are based on the same model. LL: with learning loop. noLL: without learning loop. gam: gamified. nogam: nongamified. The rugs at the bottom indicate the distribution of the measured data. Orientation scores ranged from 12 to 84. Experience scale ranged between 1 and 7.

For this research question, we focused exclusively on the mean experience (gamqMean). The main issue is whether the individual causality orientation scores (descriptive statistics in Tab.S751 (S1 File)) predict how the interface is experienced. We also sought to identify whether a change in interface would improve or worsen the experience for an individual with a given causality orientation score. For this purpose, we used a linear regression model with the interface factors of gamification and learning loop, the orientation scores for autonomy, controlled, and impersonal orientation, and the interactions terms between interface and orientation scores. The coefficients of the interaction terms answer RQ5 (main effects are described in Text S752 (S1 File)). The model explained little of the variance (R^2^ = 9%). However, the trends, shown in Fig 7, still provide some insights. They represent the predicted mean experience as a function of the causality orientation, the interface, and interactions between causality orientation and interface. We observe that some lines crossed: For autonomy-orientation scores above 65, the gamified interface with learning loop provided the best experience, and the nongamified interface without learning loop was worst. For autonomy-orientation scores below 65, the gamified interface without learning loop was best, and the nongamified interface with learning loop was worst. This complements the previous result that learning loop increased the perception of challenge (RQ2): For autonomy-oriented respondents, even if the challenge increased, the mean experience was more positive than for respondents low on autonomy orientation. The scores on controlled orientation did not influence how interface was experienced. Note, the positive slopes for the controlled-orientation scores may be exaggerated due to the higher drop-out rate of respondents with higher controlled-orientation scores. Finally, respondents with low impersonal scores tended to have a better experience with the learning loop interface than without, whereas respondents with high impersonal scores tended to have a better experience with the gamified interface with learning loop than with the nongamified interface (Fig 7).

## 4. Discussion

### 4.1. RQ1. Respondents’ participation

Our results confirm our assumptions based on the literature on online surveys [e.g., 13, 31]. More respondents abandoned the more difficult and longer survey with learning loop than the easier and shorter survey without learning loop. More respondents also dropped out of the gamified survey than the nongamified one [as in, e.g., 32, 33]. In our case, this high drop-out rate was due to a technical failure, but only partially: the unusual gamified format also raised suspicion. Some respondents did not understand why they had to choose an avatar or to enter a name, mistaking this with registration. To their mind, these seemed inappropriate to serious decision-making on wastewater management. This dovetails with results suggesting that providing preferences motivated respondents in a conjoint experiment but that gamification was not beneficial [61]. Moreover, unfamiliarity and low confidence in a gamified format [20, 62] and perceived reduced seriousness of such a format [33] previously explained lower response rate. Our gamified survey and interface with learning loop similarly lowered participation.

When market research companies are contracted to reach specific sample sizes with quotas, high drop-out rate may not directly affect the decision analysis. The sampling effort is transferred to the company. However, it might have a side-effect: If market research companies face so high drop-out rates, they may increase their fees. Furthermore, the high drop-out rate, although not problematic for the decision analysis, may bias our results. It may have filtered out the respondents on other characteristics than gender, age, and education and selected those that accept gamification (retention bias).

### 4.2. RQ2 to RQ4. Experience and basic psychological needs (BPNs)

Our results confirmed our assumptions. The interface explained very little of the experience (RQ2), particularly compared to the variance explained by the BPN (RQ3). Learning loop increased the perception of challenge as expected, while gamification increased the perception of a social experience and playfulness (RQ2). Logically, respondents who felt competent (because they mastered the given tasks) perceived less challenge (RQ3); and respondents who felt relatedness because they felt that they were connected to others perceived more social experience (RQ3). Gamification satisfied the basic psychological needs of autonomy and relatedness (RQ4), whereas the learning loop frustrated all needs (RQ4).

It is worth recalling that, although some effects are statistically significant, (1) the interpersonal variability was very large, as indicated by the low fraction of variance explained, and (2) our gamified sample might be biased due to the high drop-out rate. Overall, the interface did not clearly influence experience (RQ1), even for the very long and complex survey we tested. This is in line with previous studies observing that gamification did not improve any of their measures for engagement [14, 20, 61]. Other factors, such as the satisfaction or frustration of BPNs, seem to be more important to explaining experience. Actually, BPN satisfaction positively influenced experience. In a previous study, we found that feeling competent and volitional, which satisfied the need for autonomy, positively correlated with higher entertainment [28]. Mekler, Brühlmann, Tuch and Opwis [20] reported that satisfying autonomy and competence needs positively correlated with intrinsic motivation. The important question is thus whether the interface influences the BPNs. Our results suggest that this effect must be small. The gamified interface did not successfully satisfy all the targeted needs [20]. This stresses the relevance of investigating the effects of individual characteristics (e.g., Section 4.3). Future research should further investigate whether and how game elements can satisfy the BPNs.

### 4.3. RQ5. Individual causality orientation

Personality, as measured by the general causality orientation, seems to influence the experience. Highly autonomy-oriented respondents tended to have a better experience with the learning loop whereas respondents with lower autonomy orientation had a worse one. Knowing from RQ3 that the learning loop increased challenge and that autonomy-oriented individuals seek challenge, we suggest that highly autonomy-oriented respondents were positively challenged by the learning loop but less autonomy-oriented respondents were negatively challenged. Future studies could further explore the relations between individual causality orientation, BPN satisfaction or frustration, and challenge. The flow theory, proposing that challenge can be positive or negative [63], would be a good starting point, as it has been for some gamification studies [42]. Future studies could verify whether learning loop frustrates the BPNs of highly autonomy-oriented respondents less than of respondents with lower autonomy orientation, and how this in turn influences the perceived challenge.

Another interesting follow-up arising from the causality orientation theory is to consider gamification as a “subtle cue … to prime people’s motivational orientation” [38, p.234, S81]. Measuring the causality orientations before and after the different interfaces could verify whether an interface primes an orientation. Ideally, successful gamification would prime autonomy orientation. Priming autonomy orientation would facilitate the internalization of the external motivational affordances of the game elements, positively influencing experience and performance [20, 38].

However, we had difficulties interpreting the causality orientation because the three orientations are not mutually exclusive [38, S81]. It is also unclear how a low score in all dimensions should be interpreted. We explored the data for clusters of personality, such as a group of respondents with markedly high autonomy orientation and low impersonal and controlled orientation. However, no such clusters could be identified. Our exploratory analyses did not support any effects of age, gender, or education on any variables either, as in Mekler, Brühlmann, Tuch and Opwis [20] but unlike Koivisto and Hamari [43], who reported some age and gender effects from using a gamified app for health. The influence of individual characteristics on the perception of gamification can be investigated in many ways. We highlight only two: First, one could follow up on the Big Five personality traits [44] to confirm whether respondents scoring high on openness were attracted by and attentive to gamification and those scoring high on neuroticism had higher enjoyment with gamification. Second, one could investigate whether and how respondents liking games and gaming, either in general or only certain types of games, influences their perceptions of interface and experience [14]. Alternatively, respondents could choose between a gamified and a nongamified interface, and we could investigate the characteristics defining the two groups of respondents. Providing respondents with the choice of interface format may also confirm that if they consider the survey topic relevant, they do not need gamification [33, 61].

## 5. Conclusion

Our study rigorously evaluated the gamification of a survey for participatory public decision-making. The gamification provided a storyline connected to the survey. Our results supported most of the assumptions about gamified surveys found in the literature, sometimes nuancing them. Overall, the effect of gamifying an online survey is equivocal. Gamification tended to be associated with better experience for highly impersonal-oriented respondents and highly autonomy-oriented respondents. However, gamification led to a higher drop-out rate, possibly biasing our results. The qualitative feedback showed broad disparities in its perception. Hence, gamification is far from a “one-size-fits-all” tool. In addition, the learning loop added challenge and led to better experience for highly autonomy-oriented respondents. However, for highly impersonal-oriented respondents, the learning loop worsened the experience. Overall, the interface explained little of the variability in experience or the satisfaction and frustration of basic psychological needs. The explanatory power of the basic psychological needs on experience was much greater. Understanding this individual variability better seems paramount to making gamification beneficial to surveytainment and decision-making. Our investigation of how the general causality orientations influence the experience of various interfaces was a first step in this direction: The same game elements can benefit or hinder experience depending on the personality. Further studies could elaborate on our results, e.g. testing causal models based on structured equation modelling (SEM).

To improve gamification, further research to better understand how game elements satisfy or frustrate needs is needed. Studies could investigate individual characteristics, such as the causality orientation, or the attitude to games, given that these individual characteristics may moderate the effect of game elements on the basic psychological needs. Future studies could adopt slightly different perspectives: For instance, they could investigate if an interface primes an orientation, or they could identify characteristics of respondents choosing a specific interface when given the option to decide on the format they prefer to answer. Future studies could focus on the challenge created by learning loops: whether it is positive or negative, and for which respondents. In this study, challenge was a dimension of experience whose results differed from the other dimensions.

Overall, we have to conclude that gamification is a complex approach to increasing and improving participation that currently cannot be recommended for surveys targeting a general population: the dropout-rate is high, the improvements observed thus far are marginal, and the development costs are substantial. This does not exclude the possibility that more effective forms of gamification exist: Our results depend on the gamification we proposed and the design choices we made. Nonetheless, this study has started to disentangle the heterogeneity of responses to survey design options and thus improved our understanding of how to accommodate respondents’ diverse individual preferences. Studying gamification seems a promising research avenue for psychologists in particular.

## Supporting information

S1 File Supplementary material. This document includes all mentioned supporting information: screenshots of the gamified and control survey tool, the measurement instruments, and additional results. It starts with a table of content.
(PDF)

S1 Graphical abstract (DOCX)
