# Supplementary figures and images for: Gamified online surveys: Assessing experience with self-determination theory
Source: PLoS One. 2023 Oct 13;18(10):e0292096. doi: 10.1371/journal.pone.0292096 (PMC10575531; doi:10.1371/journal.pone.0292096)

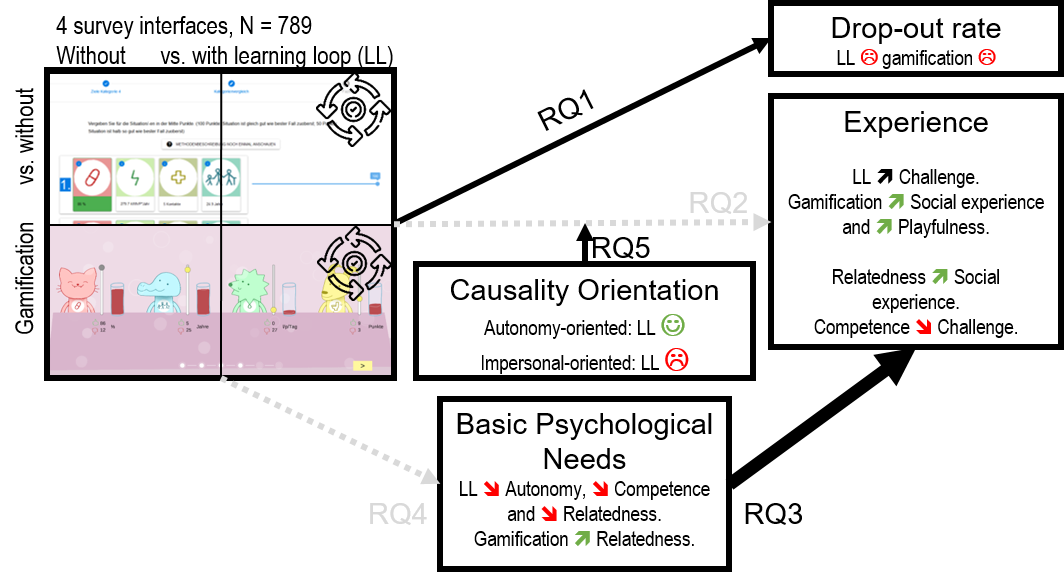

Supplement: S1 Graphical abstract — (DOCX) [file pone.0292096.s002.docx]
